# Exploring medication adherence and illness perception in patients with neuroimmune diseases: a cross-sectional study

**DOI:** 10.3389/fimmu.2026.1768709

**Published:** 2026-04-01

**Authors:** Ruizi Fu, Xiaofei Wang, Ziyan Shi, Rui Wang, Hongyu Zhou

**Affiliations:** 1 West China School of Medicine, Sichuan University, Chengdu, China; 2 Department of Neurology, West China Hospital, Sichuan University, Chengdu, China; 3 General Practice Ward, International Medical Center Ward, General Practice Medical Center, West China Hospital, Sichuan University, Chengdu, China

**Keywords:** illness perception, medication adherence, multiple sclerosis, myasthenia gravis, neuroimmune diseases, neuromyelitis optica spectrum disorder

## Abstract

**Objective:**

Neuroimmune diseases (NIDs) including myasthenia gravis (MG), multiple sclerosis (MS), and neuromyelitis optica spectrum disorder (NMOSD) require long-term medication adherence to optimize prognosis. This study aimed to investigate factors influencing medication adherence and its association with illness perception among NIDs patients.

**Methods:**

A cross-sectional survey was conducted at the Outpatient Department of Neurology, West China Hospital, Sichuan University, from March to August 2025. Patients with clinically diagnosed MG, MS, or NMOSD and ≥ 6 months of treatment were enrolled. The questionnaire on basic information, the Eight-Item Morisky Medication Adherence Scale (MMAS-8), and the Brief Illness Perception Questionnaire (BIPQ) were used for data collection. SPSS 27.0, Prism and hiplot.cn were used for statistical analysis and illustration.

**Results:**

A total of 137 valid questionnaires were collected via face-to-face interviews. Univariate analyses showed significant differences in illness perception (*F* = 43.969, *P* < 0.001), disease duration (*F* = 4.182, *P* = 0.017), and average income (*χ²* = 16.590, *P* < 0.001) across adherence subgroups. Multiple linear regression identified illness perception (*P* < 0.001) and average income (*P* = 0.003) as independent predictors, with cognitive (*P* < 0.001) and emotional representations (*P* = 0.008) exerting negative effects. Subgroup analyses revealed illness perception as the primary predictor in MG/MS patients, while income additionally affected NMOSD patients (*P* = 0.014). Low and moderate income patients were respectively influenced by emotional and cognitive representations.

**Conclusion:**

Illness perception and average income are key factors affecting medication adherence in NIDs patients, with diseases and income disparities. Individualized education targeting cognitive and emotional perceptions, along with economic considerations in treatment, may improve long-term outcomes.

## 1 Introduction

Neuroimmune diseases (NIDs) refer to a group of chronic disorders caused by abnormal immune system attacks on the nervous system, accounting for approximately 30% of the total disease burden of neurological disorders (1, 2). Among them, myasthenia gravis (MG), multiple sclerosis (MS) and neuromyelitis optica spectrum disorder (NMOSD) are the most common NIDs in China. These diseases typically have a prolonged course and are prone to recurrent episodes. Clinical prognosis relies heavily on patients’ voluntary adherence to regular medication, which is essential for controlling inflammation, preventing relapses, and delaying the progression of neurological impairments (3–5).

Medication adherence constitutes a core component in the management of chronic diseases. Studies have demonstrated that standardized treatment can help improve patients’ long-term prognosis (6). In contrast, irregular medication taking could result in poor adherence, which significantly increases the risk of relapse and may lead to irreversible neurological damage (4, 7). However, the key factors influencing medication adherence in NID patient population remained unclear.

As a critical psychocognitive factor, patients’ illness perception plays an important role in medication adherence (6). Specifically, negative illness perceptions, including overestimation of disease severity, low expectations regarding treatment efficacy, and intense emotional distress, may directly weaken patients’ willingness to take medications as prescribed. This would in turn compromise the prognosis (8, 9). Currently, the association between illness perception and medication adherence has been validated in patients with other chronic conditions (e.g., diabetes, cancers) (8, 9), but few studies have examined and validated this relationship in the context of NIDs.

Notably, distinct clinical courses and relapses patterns across NID subtypes further shape medication adherence and illness perception. For instance, MG is frequently complicated by respiratory insufficiency, a life-threatening condition that may reduce patients’ confidence in treatment and is also relatively rare in MS and NMOSD. In contrast, NMOSD more severely affects the optic nerve and spinal cord than MS, with more aggressive relapses that often lead to rapid functional decline. These disease-specific characteristics represent key factors that interacts with illness perception to influence medication adherence remain understudied.

Therefore, this study aims to investigate the associations between medication adherence and illness perception, as well as other relevant factors, in patients with NIDs. Furthermore, across different NID types, key factors influencing medication adherence are explored. Our findings are expected to provide empirical evidence for developing medication management strategies and health education interventions for patients with NIDs.

## 2 Materials and methods

### 2.1 Study population and data collection

This study adopted a cross-sectional design and was conducted at the Outpatient Department of Neurology, West China Hospital, Sichuan University, from March to August 2025. Data were consecutively collected via a questionnaire survey. The questionnaire was composed of three sections, including basic information, medication adherence, and illness perception (**Supplementary Table 1**). Additionally, the patients’ disease duration and detailed medication regimens were obtained from our database, which documents information of all NIDs patients visiting our department and has been continuously maintained for long-term clinical follow-up and research (10–12). Study purpose was fully explained to all participants and informed consent was obtained prior to questionnaire administration.

Inclusion criteria were: (1) Clinically diagnosed as MG, MS, or NMOSD according to the latest diagnostic criteria; (2) Registered in our database; (3) Had been receiving treatment for ≥ 6 months; (4) Age ≥ 16 years; (5) Able to comprehend and complete questionnaires; (6) Provided informed consent. Exclusion criteria were: (1) Other NIDs diagnosed currently or in the past; (2) Presence of other severe diseases; (3) Experience of major life events recently; (4) Failure to complete all questionnaire items; (5) The interviewer determined that the participants intentionally provided false information.

### 2.2 Questionnaire design

#### 2.2.1 Eight-item Morisky medication adherence scale

The MMAS-8 was developed by Morisky et al. as a tool for standardized assessment of medication adherence (13). It was built on prior medication adherence questionnaires, with additional incorporation of environmental influences on medication-taking behaviors to enhance its validity. Total score is calculated by summing the scores of all 8 items, with a range of 0 to 8. Higher scores indicate better medication adherence. Specifically, 8 points indicates good adherence, 6–7 points indicates moderate adherence, and a score < 6 indicates poor adherence. Based on the medication behavior patterns described in each item of the scale, we further divided the scale into three dimensions, the memory-related dimension (Items 1, 2, 4, 5), subjective perception dimension (Items 3, 6), and objective barrier dimension (Items 7, 8).

#### 2.2.2 The brief illness perception questionnaire

The BIPQ was developed by Broadbent et al. It is a self-reported questionnaire consisting of nine items, which comprehensively and objectively reflects patients’ perception of their illness (14). Five items evaluate cognitive illness representations, including consequences, timeline, personal control, treatment control, and identity. Two items evaluate emotional illness representations, including concern and emotional response. One item evaluates illness comprehension. All items are scored using a 0 to 10 response scale, with items 3 (personal control), 4 (treatment control), and 7 (illness comprehensibility) scored in reverse. Higher total scores indicate stronger negative perceptions.

Both questionnaires have undergone reliability and validity evaluations in China.

### 2.3 Statistical analysis

All data collected in this study were analyzed using SPSS 27.0. Results of correlation analysis and forest maps were illustrated using Prism, while inter-group comparisons of dimensions of illness perception and heatmaps were illustrated via https://hiplot.cn. Continuous variables were presented as Mean ± SD, and inter-group comparisons were performed using one-way ANOVA. Categorical variables were presented as counts and percentages, with inter-group comparisons performed using the χ² test. Spearman’s correlation analysis was applied to explore the association between medication adherence and illness perception as well as their respective dimensions. Multiple linear regression analysis was performed to further identify factors potentially influencing medication adherence. Additionally, subgroup analyses were conducted to explore differences in the factors affecting medication adherence among patients with different NIDs or different income levels. Receiver operating characteristic (ROC) curves were constructed for both questionnaires across the three NID subgroups. P<0.05 was considered statistically significant.

## 3 Results

### 3.1 Basic information for study population

Questionnaire data were collected via face-to-face surveys. A total of 137 valid questionnaires were confirmed after random screening of patients with a definite diagnosis of MG, MS and NMOSD from our database. Demographic characteristics of the participants are presented in **Table 1**.

**Table 1:** Demographic characteristics among disease groups.

Characteristics	All (n=137)	MG (n=83)	MS (n=16)	NMOSD (n=38)
Gender (n,%)				
Male	44 (32.1)	35 (42.2)	4 (25.0)	5 (13.2)
Female	93 (67.9)	48 (57.8)	12 (75.0)	33 (86.8)
Age (Mean ± SD)	43.15 ± 16.07	44.25 ± 16.16	29.19 ± 8.90	46.61 ± 14.45
Medication (n,%)				
Immunosuppressants^a^	30 (21.9)	15 (18.1)	0 (0)	15 (39.5)
Monoclonal Antibodies^b^	26 (19.0)	5 (6.0)	5 (31.3)	16 (42.1)
Other Drugs^c^	33 (24.1)	22 (26.5)	11 (68.8)	0 (0)
Not in Use	48 (35.0)	41 (49.4)	0 (0)	7 (18.4)
Education (n,%)				
High School or Below	76 (55.5)	43 (51.8)	5 (31.3)	28 (73.7)
Above High School	61 (44.5)	40 (48.2)	11 (68.8)	10 (26.3)
Household size (n,%)				
Living Alone	13 (9.5)	7 (8.4)	3 (18.8)	3 (7.9)
Living with 2–5 People	112 (81.8)	71 (85.5)	11 (68.8)	30 (78.9)
Living with More Than 5 People	12 (8.8)	5 (6.0)	2 (12.5)	5 (13.2)
Residential area (n,%)				
Urban	77 (56.2)	45 (54.2)	11 (68.8)	21 (55.3)
Town	34 (24.8)	22 (26.5)	2 (12.5)	10 (26.3)
Rural	26 (19.0)	16 (19.3)	3 (18.8)	7 (18.4)
Average income, month (n,%)				
≤ ¥3000	45 (32.8)	27 (32.5)	6 (37.5)	12 (31.6)
>¥3000	92 (67.2)	56 (67.5)	10 (62.5)	26 (68.4)
Insurance (n,%)				
No Insurance Purchased	11 (8.0)	8 (9.6)	0 (0)	3 (7.9)
Mandatory National Insurance	116 (84.7)	71 (85.5)	15 (93.8)	30 (78.9)
Commercial Insurance	10 (7.3)	4 (4.8)	1 (6.3)	5 (13.2)
Disease Duration, month (Mean ± SD)	62.52 ± 58.35	68.80 ± 66.40	62.38 ± 40.21	48.87 ± 42.49
Medication Adherence Score (Mean ± SD)	6.58 ± 1.49	6.48 ± 1.55	6.48 ± 1.40	6.84 ± 1.39
Total Illness Perception Score (Mean ± SD)	48.78 ± 9.81	49.00 ± 9.81	49.38 ± 9.34	48.05 ± 10.24

^a^Immunosuppressants: Mycophenolate Mofetil, Azathioprine, Tacrolimus; ^b^Monoclonal Antibodies: Rituximab, Inebilizumab, Satralizumab; ^c^Other Drugs: Prednisone, Pyridostigmine etc.

**Figure 1:**
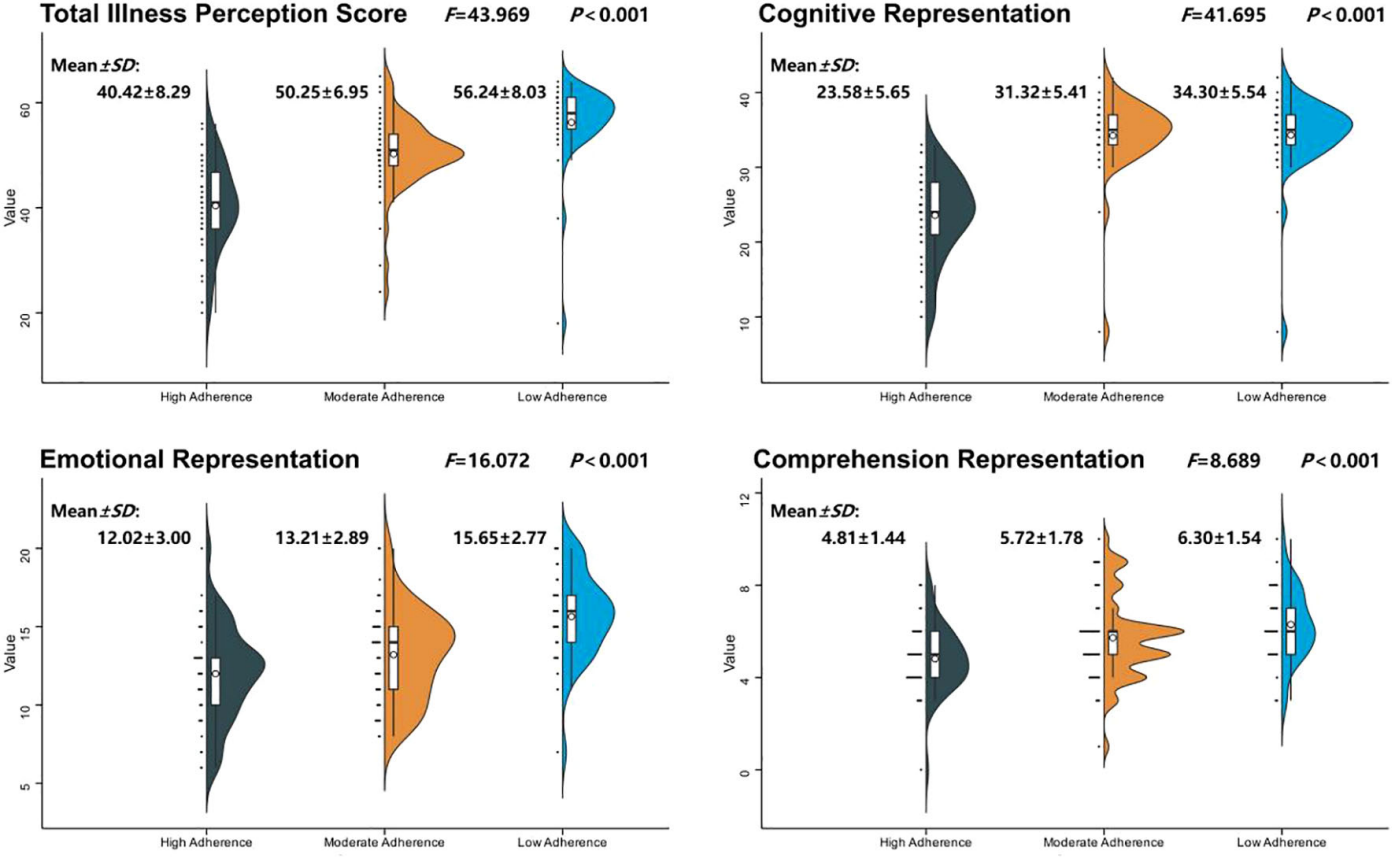
Illness perception scores and dimensions among adherence groups.

We stratified participants into three groups according to tertiles based on BIPQ scores, namely, the high adherence group (n=43), moderate adherence group (n=57), and low adherence group (n=37). Demographic characteristics and illness perception scores were compared across the three groups. Illness perception (*F* = 43.969, *P* < 0.001), disease duration (*F* = 4.182, *P* = 0.017), and average income (*χ²* = 16.590, *P* < 0.001) were significantly different among groups. Detailed results are presented in **Table 2** and **Figure 1**.

**Table 2:** Demographic characteristics among adherence groups.

Characteristics	High adherence (n=43)	Moderate adherence (n=57)	Low adherence (n=37)	Statistics	*P*
Gender (n,%)					
Male	17 (39.5)	14 (24.6)	13 (35.1)	*χ²=*2.732	0.255
Female	26 (60.5)	43 (75.4)	24 (64.9)		
Age (Mean ± SD)	44.70 ± 16.77	41.51 ± 14.93	43.86 ± 17.12	*F* = 0.530	0.590
Disease type (n,%)					
MG	27 (62.8)	29 (50.9)	27 (73.0)	*χ²=*7.814	0.099
MS	2 (4.7)	10 (17.5)	4 (10.8)		
NMO	14 (32.6)	18 (31.6)	6 (16.2)		
Medication (n,%)					
Immunosuppressants^a^	11 (25.6)	11 (19.3)	8 (21.6)	*χ²* = 3.072	0.800
Monoclonal Antibodies^b^	7 (16.3)	13 (22.8)	6 (16.2)		
Other Drugs^c^	9 (20.9)	12 (21.1)	12 (32.4)		
Not in Use	16 (37.2)	21 (36.8)	11 (29.7)		
Education (n,%)					
High School or Below	24 (55.8)	33 (57.9)	19 (51.4)	*χ²* = 0.392	0.822
Above High School	19 (44.2)	24 (42.1)	18 (48.6)		
Household size (n,%)					
Living Alone	5 (11.6)	4 (7.0)	4 (10.8)	*χ²=*1.070	0.899
Living with 2–5 People	35 (81.4)	48 (84.2)	29 (78.4)		
Living with More Than 5 People	3 (7.9)	5 (8.8)	4 (10.8)		
Residential area (n,%)					
Urban	23 (53,5)	35 (61.4)	19 (51.4)	*χ²* = 2.298	0.681
Town	11 (25.6)	11 (19.3)	12 (32.4)		
Rural	9 (20.9)	11 (19.3)	6 (16.2)		
Average income, month (n,%)					
≤ ¥3000	6 (14.0)	18 (31.6)	21 (56.8)	*χ²* = 16.590	*<0.001*
>¥3000	37 (86.0)	39 (68.4)	16 (43.2)		
Insurance (n,%)					
No Insurance Purchased	2 (4.7)	5 (8.8)	4 (10.8)	*χ²* = 1.896	0.755
Mandatory National Insurance	39 (90.7)	47 (82.5)	30 (81.1)		
Commercial Insurance	2 (4.7)	5 (8.8)	3 (8.1)		
Disease Duration, month (Mean ± SD)	50.23 ± 44.04	57.07 ± 52.59	85.19 ± 74.43	*F* = 4.182	*0.017*

^a^Immunosuppressants: Mycophenolate Mofetil, Azathioprine, Tacrolimus; ^b^Monoclonal Antibodies: Rituximab, Inebilizumab, Satralizumab; ^c^Other Drugs: Prednisone, Pyridostigmine etc.

### 3.2 Spearman’s correlation analysis

**Figure 2:**
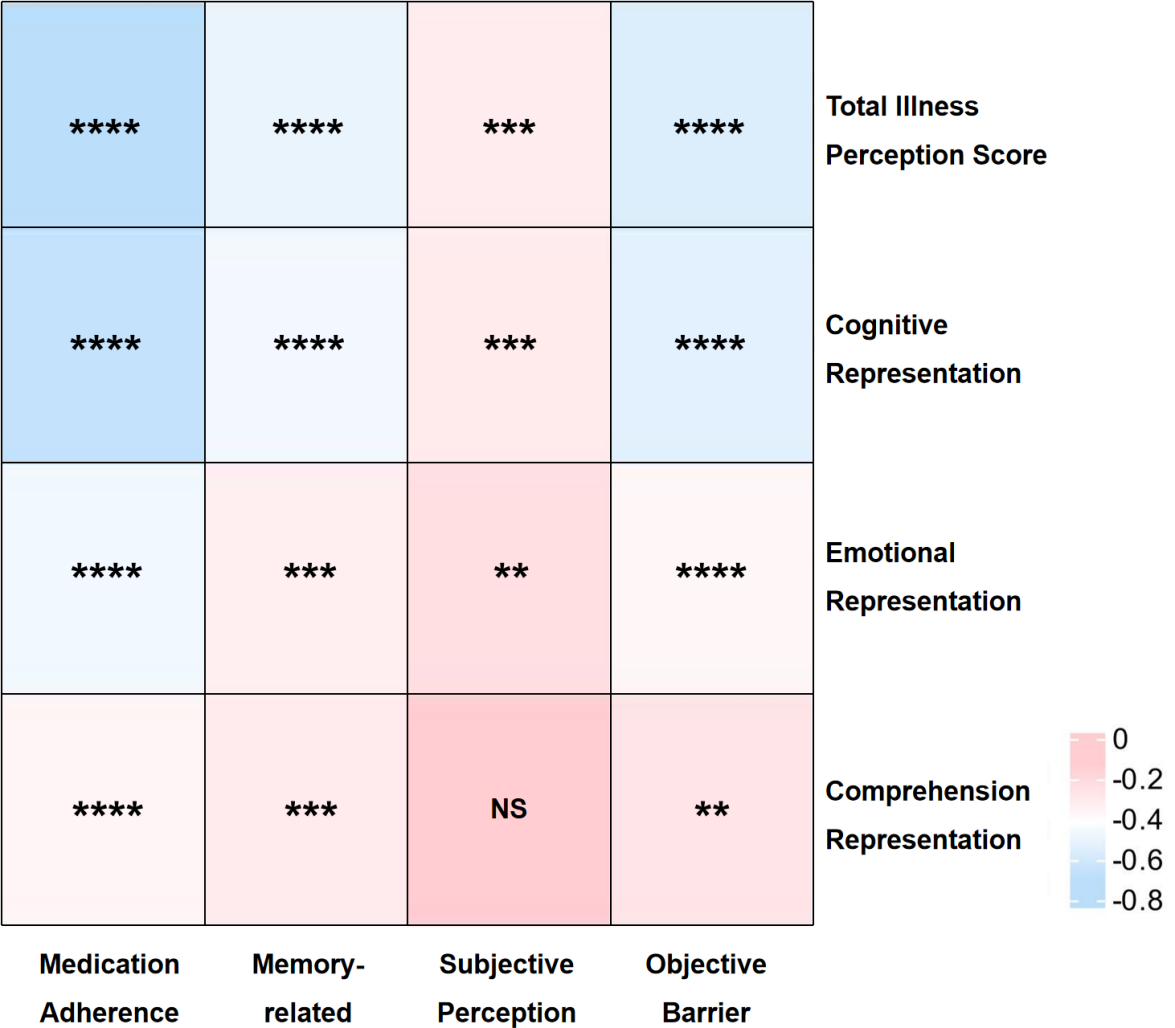
Associations of Illness Perception and Medication Adherence; * P < 0.05; ** P < 0.01; *** P < 0.001; **** P < 0.001; NS, Not Significant.

We performed Spearman’s correlation analysis to examine the associations between medication adherence and illness perception as well as their respective dimensions. We found no significant correlations between patients’ subjective perceptions of medication use and their comprehension representation, while all other dimensions were significantly negatively correlated (**Figure 2** and **Supplementary Figure 1**).

### 3.3 Multiple linear regression analysis

**Figure 3:**
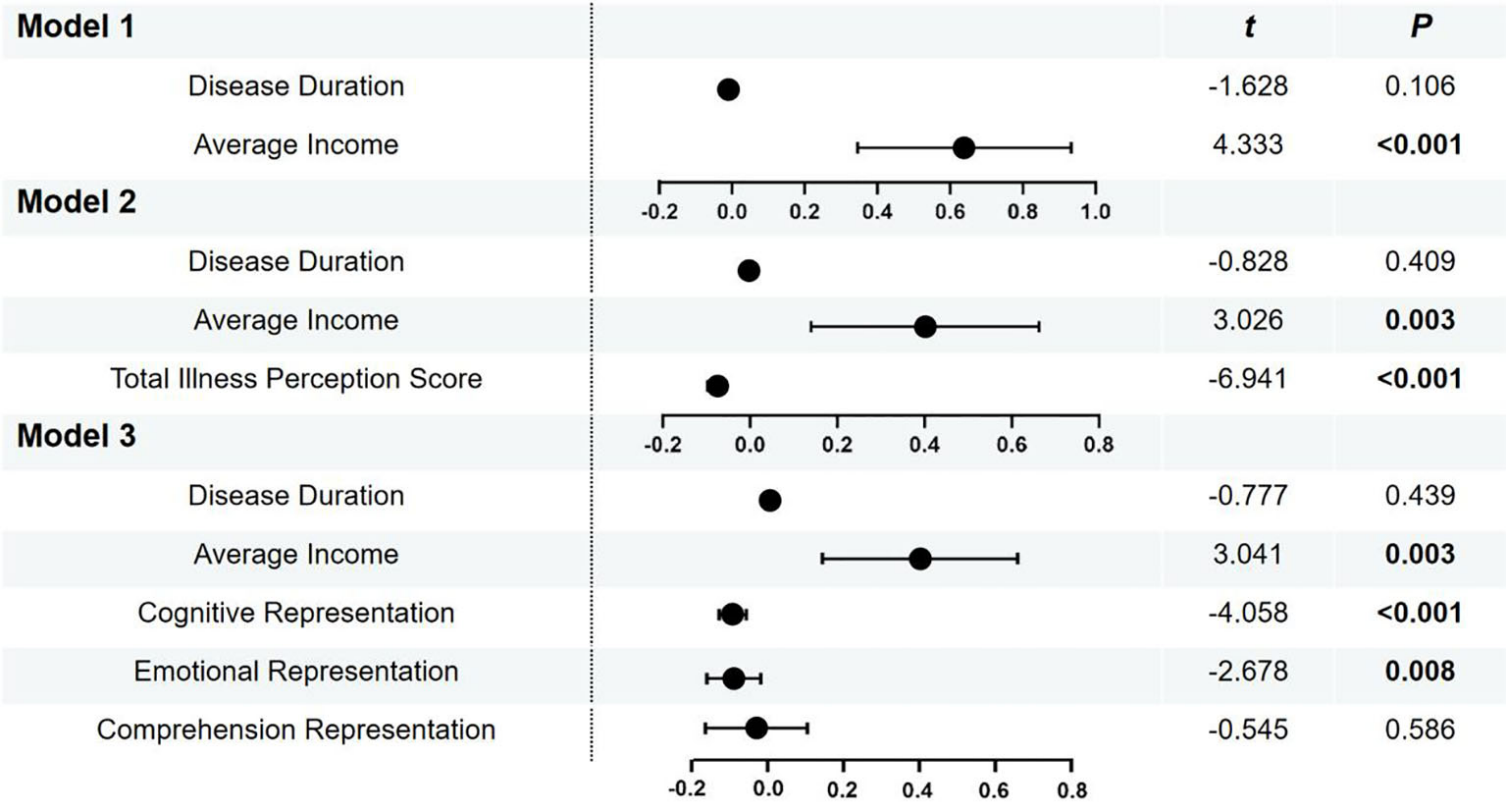
Multiple linear regression results.

We performed multiple linear regression analysis to assess the associations between medication adherence score and the statistically significant influencing factors from **Table 2** and illness perception. We further categorized average monthly income into three groups using cut-off values of 3000 yuan and 6000 yuan. Disease duration and average income were included in the model as control variables in Model 1, followed by the total illness perception score as the core independent variable in Model 2. The three dimensions of illness perception respectively served as subdivided independent variables in Model 3. We found that patients’ average income and total illness perception score were significant influencing factors of medication adherence. Among them, the cognitive representation and emotional representation exerted a statistically significant negative impact on adherence. Detailed results are presented in **Figure 3**.

We performed multiple linear regression analyses with each of the three dimensions of medication adherence as the dependent variable, and the statistically significant influencing factors from **Table 2**, as well as illness perception and its dimensions, as the independent variables. We found that average income and cognitive representation in illness perception had significant effects on memory-related dimension and objective barrier. Detailed results are presented in **Supplementary Figure 2**. Collinearity diagnosis indicated that all variance inflation factors (VIF) were < 5.

### 3.4 Subgroup analysis

#### 3.4.1 Subgroup analysis based on NID types

We performed multiple linear regression analysis with the total medication adherence score as the dependent variable, and disease duration, average income, and total illness perception score as independent variables. The results showed that in the MG and MS subgroups, the total illness perception score had a significant negative impact on medication adherence, while disease duration and average income level exerted no statistically significant effects. In the NMOSD subgroup, average income level and total illness perception score had significant positive and negative impacts on medication adherence, respectively, whereas disease duration showed no significant influence. Detailed results are presented in **Supplementary Figure 3**.

Furthermore, we introduced the three dimensions of illness perception into the model as independent variables. We found that the cognitive representation of illness perception exerted a negative impact on medication adherence in both MG and MS subgroups, while the MG subgroup was additionally influenced by emotional representation. On the other hand, only the NMOSD subgroup was significantly negatively affected by average income. Detailed results are presented in **Supplementary Figure 4**.

#### 3.4.2 Subgroup analysis based on average income

We performed multiple linear regression analysis with the total medication adherence score as the dependent variable, and disease duration and total illness perception score as independent variables. We found that the total illness perception score had a significant negative impact on medication adherence in all subgroups. Detailed results are presented in **Supplementary Figure 5**.

Furthermore, we introduced the three dimensions of illness perception into the model as independent variables. The results indicated that the cognitive representation and emotional representation of illness perception exerted a significant negative impact on the moderate income group and low income group respectively. No significant effect of any dimension was observed in the high income group. Detailed results are presented in **Supplementary Figure 6**.

### 3.5 ROC curve analysis

We performed ROC curve analysis to compare the sensitivity, specificity, positive and negative predictive values for medication adherence in the NID subgroups. Area Under the Curve (AUC) for MG, MS and NMOSD subgroup were respectively 0.899, 0.865 and 0.760. Negative Predictive Values (NPVs) for the three subgroups were respectively 0.735, 0.571 and 0.312. Positive Predictive Values (PPVs) for the three subgroups were respectively 0.959, 1.000 and 0.955, indicating high credibility of positive predictions in each subgroup. Detailed results are presented in **Supplementary Table 2** and **Supplementary Figure 7**.

### 3.6 Reliability and criterion validity

For MMAS-8, the Cronbach’s α coefficient in this study were respectively 0.60. Among the disease subgroups, those for MG, MS, and NMOSD were respectively 0.62, 0.47 and 0.60. For BIPQ, the Cronbach’s α coefficient in this study were respectively 0.82. Among the disease subgroups, those for MG, MS, and NMOSD were respectively 0.83, 0.81 and 0.81.

Furthermore, the criterion validity were addressed by assessing the correlation between factors validated in previous studies and, respectively, medication adherence and illness perception. In the multiple linear regression, questionnaire scores were applied as the dependent variables. Detailed results are presented in **Supplementary Tables 3**, **4**.

## 4 Discussion

Medication adherence is a key factor determining the disease control effect and prognosis in long-term treatment of NIDs. Our cross-sectional study aimed to explore the factors influencing medication adherence and the associations with illness perception among patients with MG, MS and NMOSD. Our results revealed that average income, disease duration, and illness perception had negative impacts on medication adherence. A lower level of negative illness perception indicated better medication adherence. Multiple linear regression analysis further demonstrated that illness perception and average income were independent predictors of medication adherence. Subgroup analysis suggested distinctive features between diseases. Among MG and MS patients, total illness perception score was the primary negative predictor of medication adherence, whereas among NMOSD patients, in addition to illness perception, average income also emerged as a significant influencing factor. Subgroup analysis by income level suggested that patients with different income levels might be affected by different dimensions of illness perception.

Our findings are consistent with previous studies on chronic diseases, together confirming that patients’ illness perception is a key factor influencing medication adherence. Patients’ cognitive and emotional responses to their illness can directly affect their treatment behaviors. In particular, negative illness perceptions may diminish medication-taking motivation (6, 9). Broadbent et al. and Eshete et al. both demonstrated in patients with diabetes that positive illness perceptions effectively improve medication adherence (15, 16). Similarly, Liu et al. and Chen et al. respectively validated the association between illness perception and medication adherence in patients with chronic obstructive pulmonary disease and colorectal cancer (17, 18). Along with existing evidence, our results illustrate that illness perception, as patients’ own representations of the diseases, shapes their treatment behaviors and holds important implications for long-term management (19).

The three dimensions of illness perception in this study showed significant heterogeneity in their impact on medication adherence. Among them, cognitive representation showed the most prominent negative predictive effect. This result aligns with the core proposition of the Common Sense Model of Illness Self-Regulation, that is, an individual’s cognitive representation of illness is the core mediating mechanism driving health behavior decisions, directly reflecting patients’ trade-off between medication necessity and disease threat. In contrast, emotional and comprehension representations are at different levels of behavior regulation respectively (20, 21). Also, from the perspective of the Necessity-Concerns Framework, if patients perceive that the disease has a greater impact on their health, their motivation to avoid health risks through regular medication is stronger. Conversely, they may mistakenly regard medication as an unnecessary behavior, leading to lower adherence (6, 22). By comparison, the comprehension representation showed no statistical significance in all models. This may relate to the complexity of the chronic NIDs studied. Their pathogenesis and therapeutic rationale are highly specialized, making it difficult for patients to comprehend such knowledge, which hinders them from grasping the intrinsic link between medication and personal health, precluding the formation of effective medication motivation, and thereby weakening the impact on adherence.

Beyond the primary findings, distinct characteristics of illness perception across MG, MS and NMOSD further elaborate the heterogeneity of medication adherence. As suggested by the subgroup results, cognitive representation extorted a prominent negative effect on adherence among MG patients, whereas its impact was non-significant in NMOSD. As mentioned before, the life-threatening respiratory insufficiency and myasthenic crisis may reinforce negative evaluation of treatment efficacy (5). Similarly, MS often presents as relapsing-remitting courses with neurological impairments, which may also undermine patients’ trust and reduce long-term adherence (7). On the contrary, NMOSD mainly manifested as acute optic neuritis and myelitis, shifting patients’ cognitive attention to symptom control in acute phase and ultimately making long-term perceptions less influential (4, 23). Likewise, the emotional representation was only significant in MG subgroup, which may be attributed to its higher risk of severe complications that induces persistent distress.

Additionally, dosage regimens and adverse effects mediate the relationship between medication adherence and illness perception in different ways. Nowadays, MG and MS predominantly use oral disease-modifying drugs (DMDs) with flexible administration, which can reduce treatment burden to some extent (5, 7). However, NMOSD relies more heavily on monoclonal antibodies (23). In this scenario, the infusion requirements, higher costs and stricter dosing schedules may all reinforce negative perceptions of treatment accessibility and compromises medication adherence. Adverse effects further amplify aforementioned association, in that long-term use of corticosteroids in MG may cause weight gain or infections, while oral DMDs in MS may induce fatigue or gastrointestinal discomfort (5). These observations are consistent with the Necessity-Concerns Framework, which posits that when patients perceive the risks of medication taking as outweighing its potential benefits, it tends to results in medication non-adherence (6, 24).

On the other hand, average income was significantly associated with medication adherence in both the overall sample and the NMOSD subgroup, suggesting that economic burden may be a key influencing factor in the long-term management of NIDs. Babazadeh et al. revealed a significant correlation between low income and poor medication adherence, while Agh et al. also noted that economic hardship directly threatens medication accessibility and continuity (9, 25). Amid the high costs, complex courses and rare nature of NIDs, economic burden may be more pronounced, thereby amplifying its impact on medication adherence (26, 27). However, in the MG and MS subgroups, average income had no significant impact.

This discrepancy may stem from distinct disease characteristics, treatment regimens as well as other factors, leading to variations in medication behaviors (26, 28, 29). Among patients enrolled in this study, 42.1% of NMOSD patients were receiving monoclonal antibody therapy, a proportion significantly higher than that in MG patients (6.0%) and MS patients (31.3%). Monoclonal antibodies are more costly in China, making NMOSD patients’ medication behaviors more susceptible to economic burden and thus rendering average income an independent predictor of adherence (4, 23, 30).

From a socioeconomic perspective, China’s healthcare system and insurance policies may be a key factor in shaping patients’ illness perception and medication adherence among NID patients. China’s multi-tiered medical insurance framework covers conventional medications to a certain extent, for instance, oral DMDs for MG and MS. However, for high-costs therapies such as monoclonal antibodies in NMOSD, it provides limited reimbursement (31, 32). This coverage disparity directly influences illness perception. While MG and MS patients with more accessible oral medications hold relatively positive perceptions of treatment, NMOSD patients may face heavier expenses, thus tend to develop negative cognitive representations of disease burden (4, 11), Insufficient coverage may lead patients’ to prioritize short-term economic concerns over long-term management, forming negative illness perceptions that may reduce adherence (25, 27, 33).

In subgroup analysis by average income, there were also differences in the impact of illness perception dimensions among different subgroups, which may be attributed to restriction of economic level on health decision-making. The low income subgroup was significantly affected by emotional representation, possibly because the economic burden of treatment easily translates disease-related negative emotions into resistance to medication. Meanwhile, the moderate income subgroup had a relatively alleviated economic burden, as a result, they paid more attention to rational cognition such as medication necessity (6, 34). In the high income subgroup, no prominent impact of any dimension was observed, which may be because economic advantages offset the differences among the dimensions of illness perception to some extent, making their medication behavior relatively unrestricted.

While exploring the factors impacting medication adherence, it is also worth considering whether high adherence is universally indispensable for NIDs, as oral DMDs have achieved substantial relapse reduction in MG and MS despite median adherence rates of only 65-70% (5, 7). However, though higher adherence is not indispensable for clinical benefit, incremental improvements in adherence still correlate with better outcomes such as reduced cognitive decline (33). Non-adherence in NIDs often arises from modifiable factors including medication burden, adverse effects and socioeconomic constraints, emphasizing that interventions should prioritize personalized treatment plan rather than rigid adherence targets (9, 32, 35).

This study has several limitations. First, the cross-sectional design cannot make inference of causal relationships. Future studies could adopt longitudinal designs or intervention trials based on our database to validate the dynamic association between illness perception and medication adherence (36). Second, the single-center sample may introduce selection bias, limiting the external validity to some extent. Additionally, the MMAS-8 exhibited low internal consistency reliability in this study, though the value remained within the range reported in previous relevant researches (37–39). This may be primarily attributed to the dichotomous scoring pattern of the scale. Furthermore, potential inconsistency in the content of questionnaire items may have affected patients’ understanding, leading to low internal consistency of the results. Notably, in the MS subgroup, the Cronbach’s α coefficient of MMAS-8 failed to meet the acceptable criterion, suggesting that this scale may not be applicable to this population. Notably, though factors including education level and medication heterogeneity were included in the initial analysis, they did not show statistical significance in group comparisons, which may confound the association between illness perception and medication adherence.

In summary, this study demonstrates that illness perception is significantly associated with medication adherence among patients with NIDs, using data from our database covering all types of NIDs. It may provide direction for future clinical practice. For instance, routine assessment of illness perception could be integrated into outpatient visits and database follow-up. In order to correct cognitive biases and enhance treatment confidence, individualized health education should be tailored to patients who showed pronounced negative illness perceptions. For the cognitive representation, a series of patient-oriented health education programs can be designed to help patients better understand their own diseases in an accessible manner to foster medication motivation. Likewise, clinicians should fully consider patients’ economic status when deciding treatment plans. In addition, patients in China with a monthly income of ≤ 3000 yuan may face significant difficulties in medication access. Therefore, when developing plans for this group, their affordability should be taken into account to prevent economic constraints from affecting these patients’ adherence and even long-term prognosis. Future research could explore the feasibility of intervention strategies based on eHealth, with the aim to improve the medication adherence and introduce new insights in NIDs patients’ management (35).

## Data Availability

The raw data supporting the conclusions of this article will be made available by the authors, without undue reservation.
