# Supplementary material for: Exploring medication adherence and illness perception in patients with neuroimmune diseases: a cross-sectional study
Source: Front Immunol. 2026 Apr 1;17:1768709. doi: 10.3389/fimmu.2026.1768709 (PMC13079056; doi:10.3389/fimmu.2026.1768709)
Supplement: Supplementary file 1 [file DataSheet1.docx]

Supplementary Material

# Supplementary Figures

**Supplementary Figure 1** Results of Spearman’s Correlation Analysis

**Supplementary Figure 2** Multiple Linear Regression Results in Dimensions of Medication Adherence

**Supplementary Figure 3** Multiple Linear Regression Results in Disease Subgroups with Total Illness Perception Score

**Supplementary Figure 4** Multiple Linear Regression Results in Disease Subgroups with Dimensions of Illness Perception

**Supplementary Figure 5** Multiple Linear Regression Results in Income Subgroups with Total Illness Perception Score

**Supplementary Figure 6** Multiple Linear Regression Results in Income Subgroups with Dimensions of Illness Perception

**Supplementary Figure 7** ROC Curves for MG, MS and NMOSD Subgroups


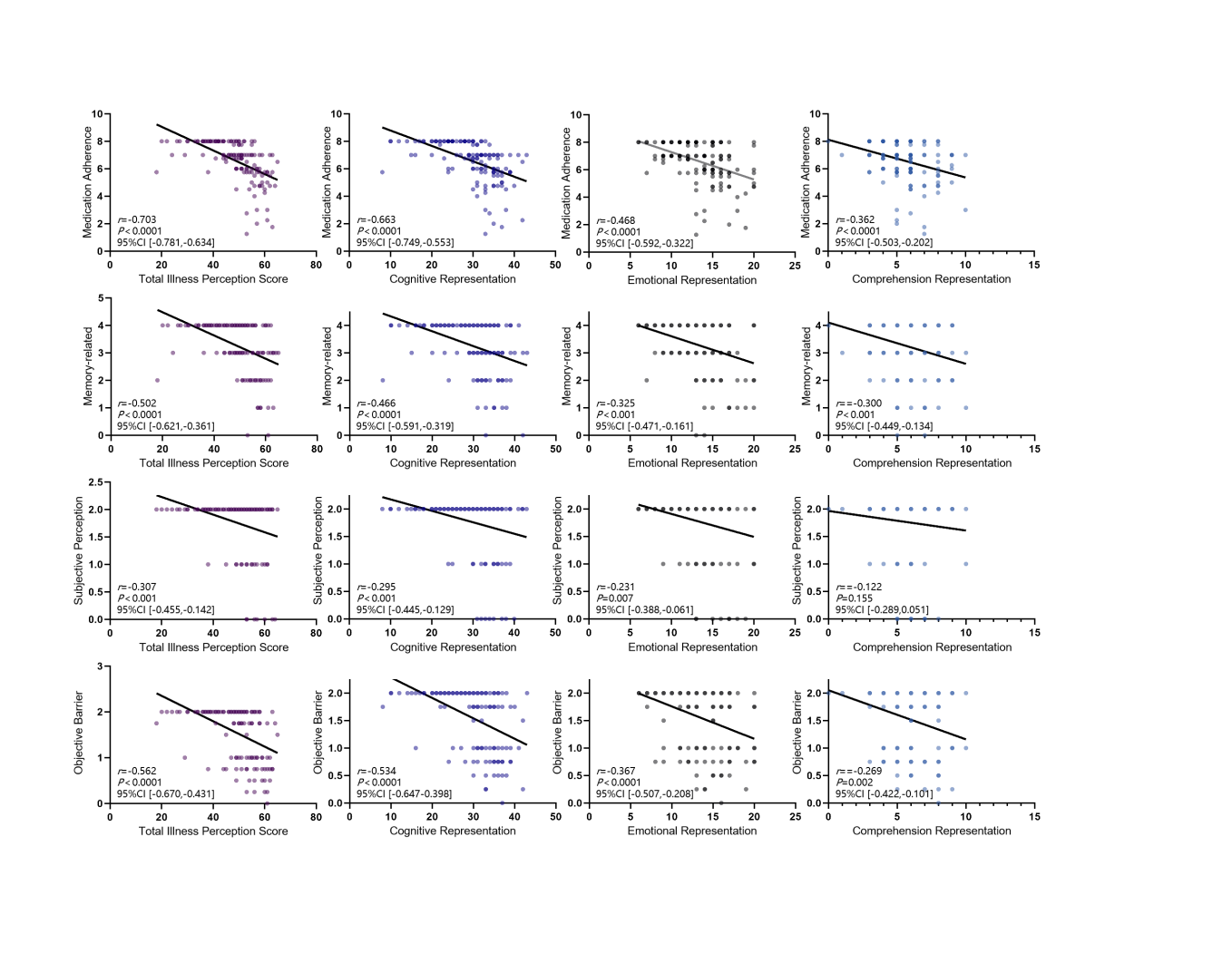


**Supplementary Figure 1** Results of Spearman’s Correlation Analysis


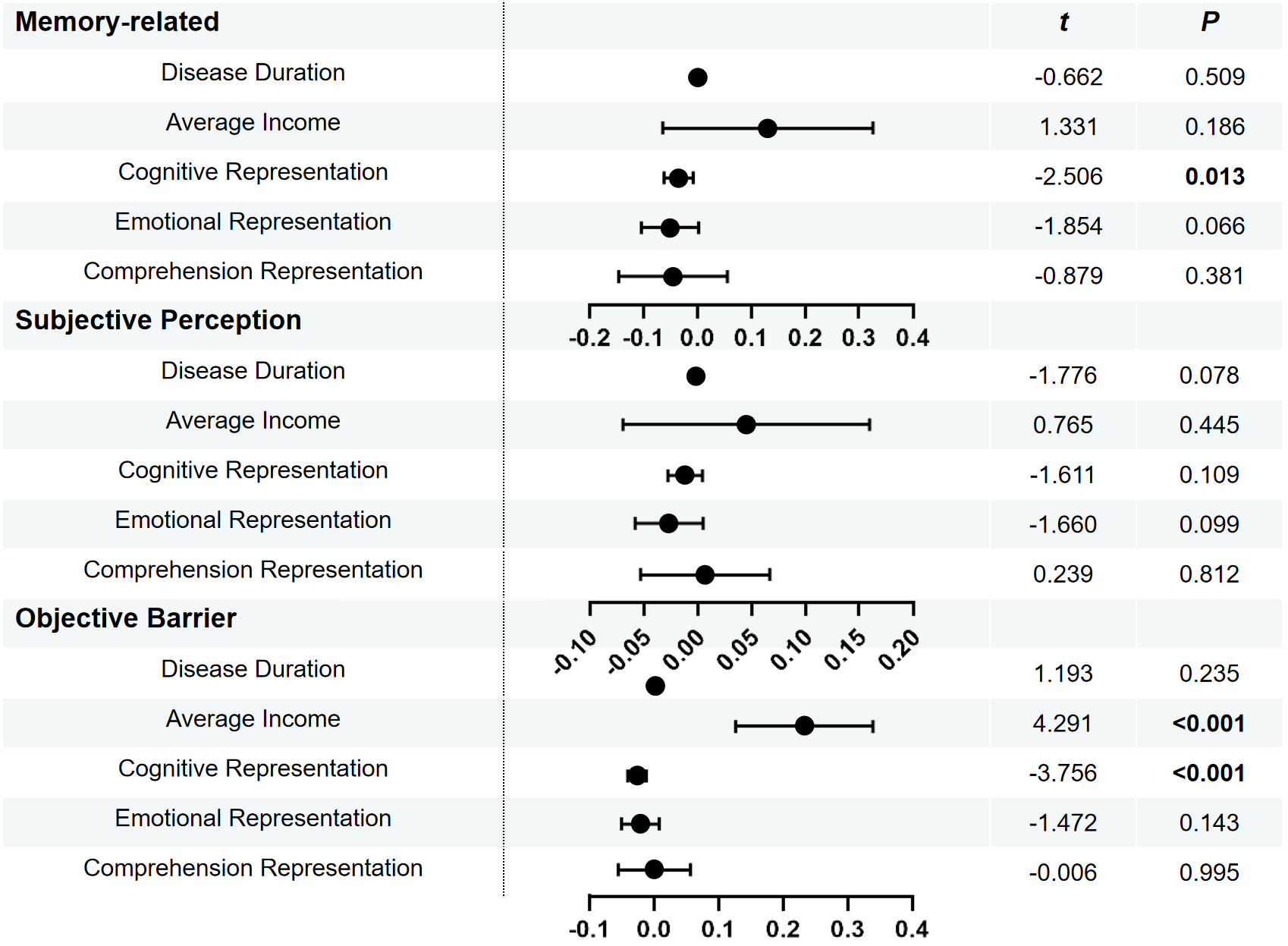


**Supplementary Figure 2** Multiple Linear Regression Results in Dimensions of Medication Adherence


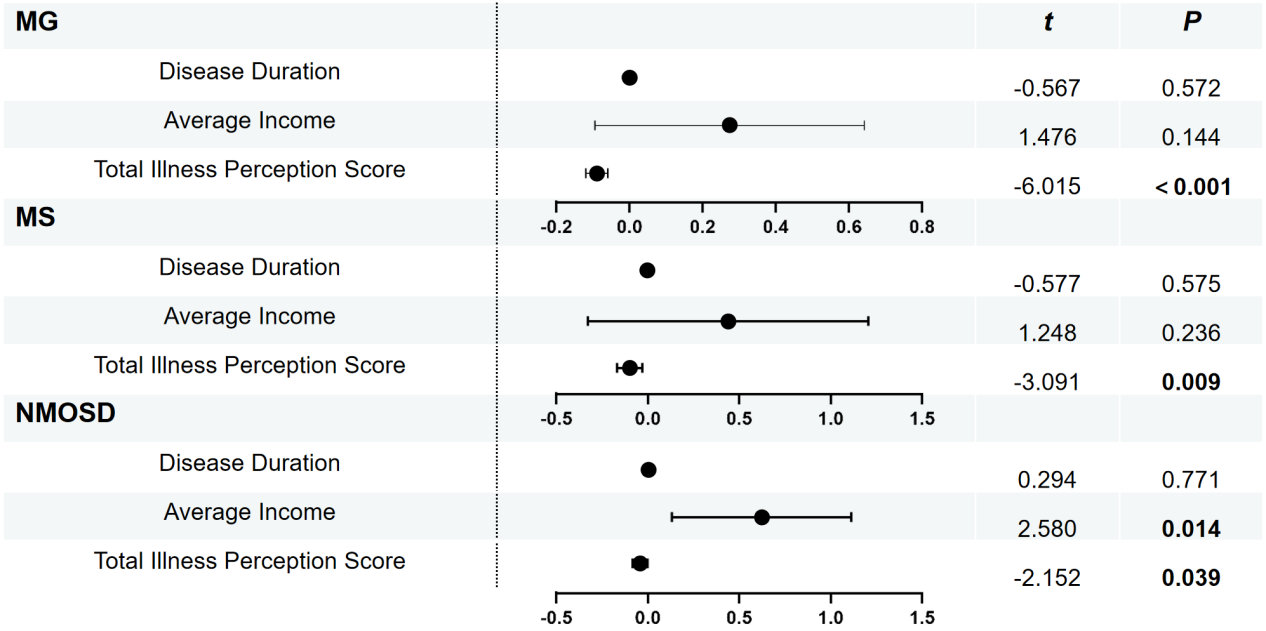


**Supplementary Figure 3** Multiple Linear Regression Results in Disease Subgroups with Total Illness Perception Score

is


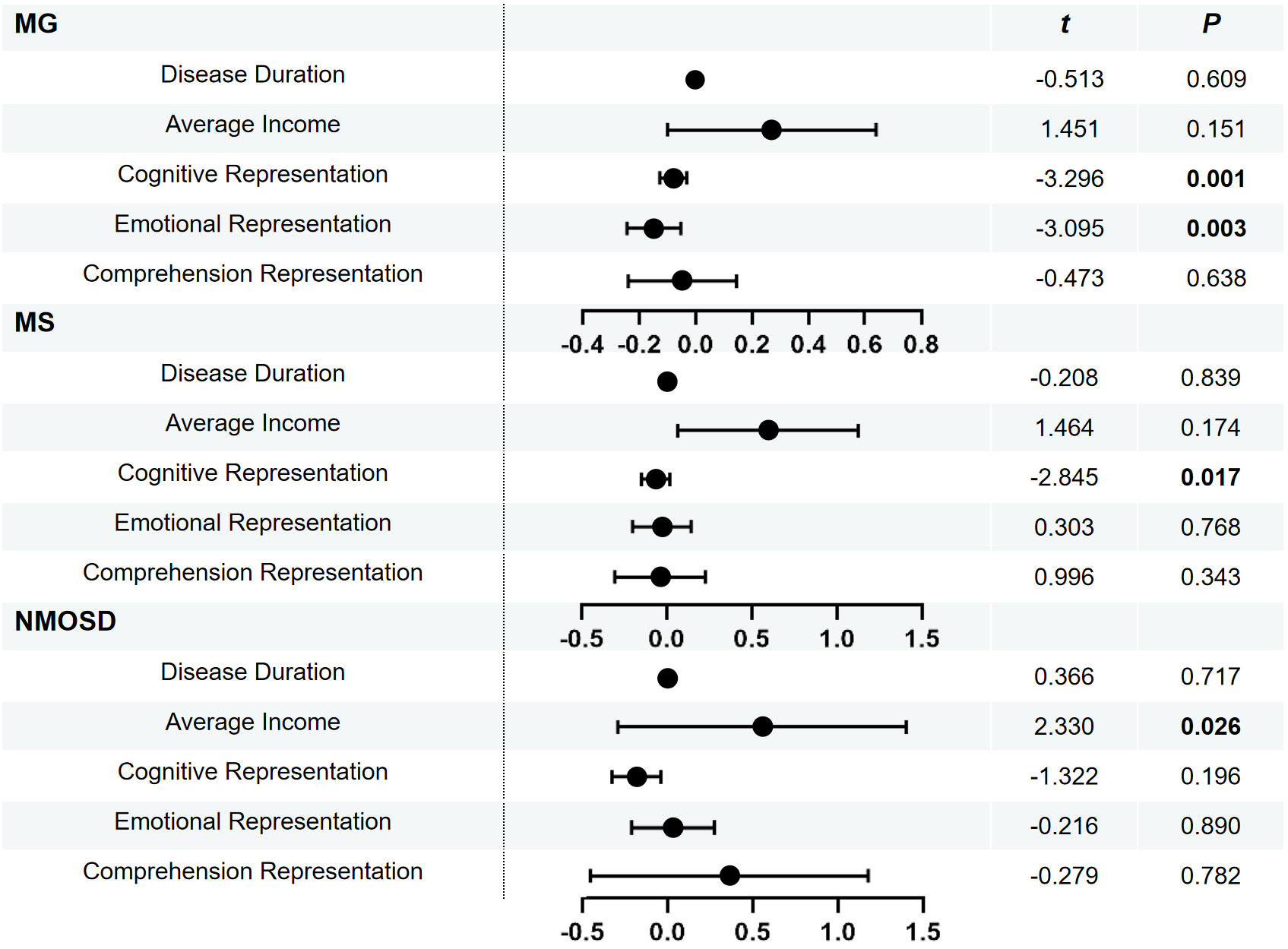


**Supplementary Figure 4** Multiple Linear Regression Results in Disease Subgroups with Dimensions of Illness Perception


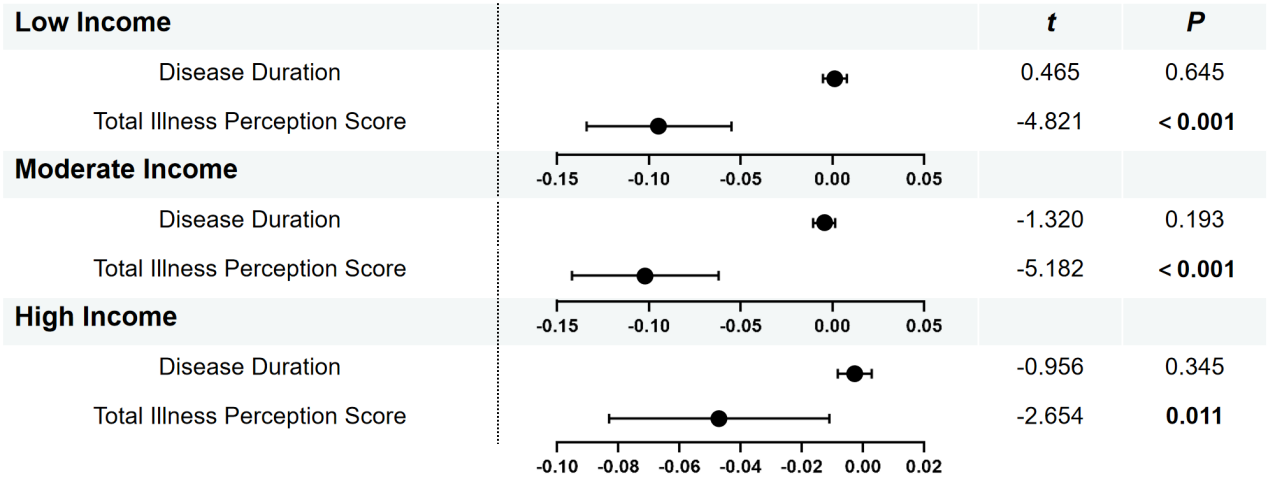


**Supplementary Figure 5** Multiple Linear Regression Results in Income Subgroups with Total Illness Perception Score


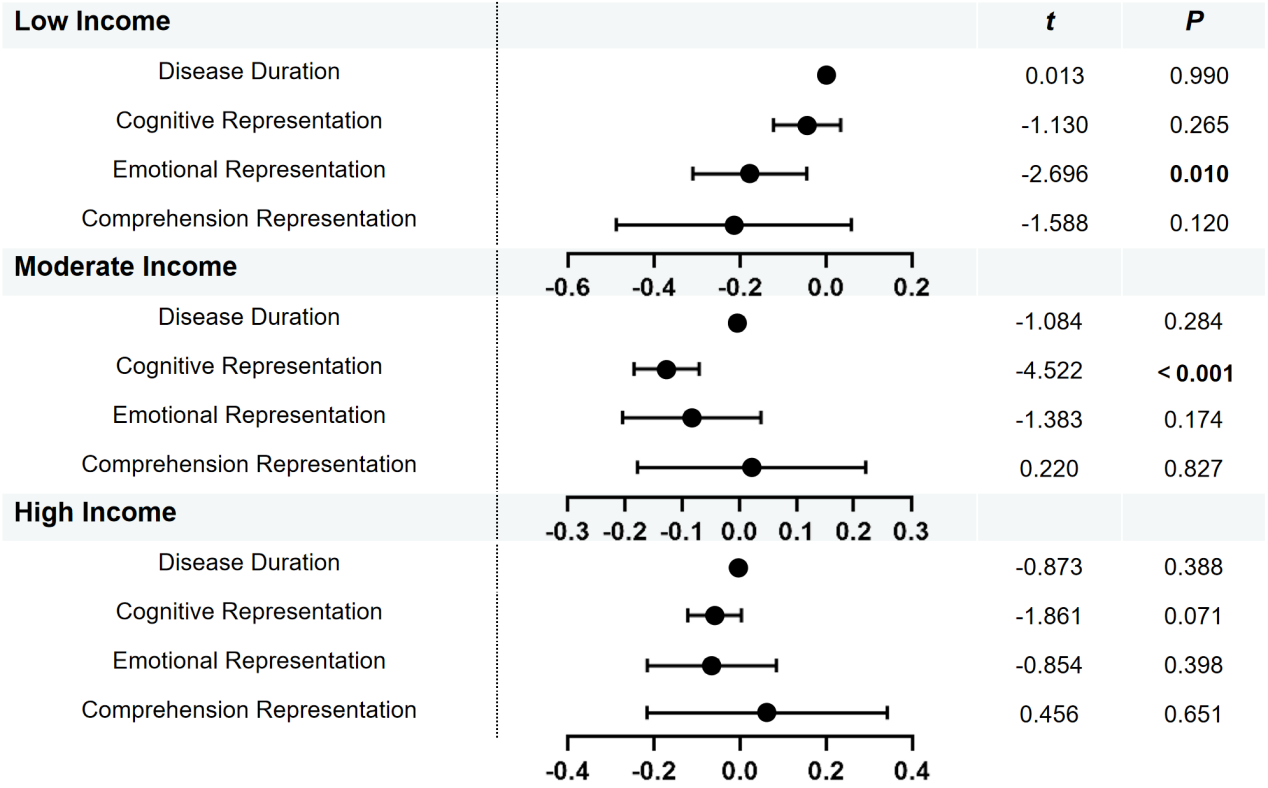


**Supplementary Figure 6** Multiple Linear Regression Results in Income Subgroups with Dimensions of Illness Perception


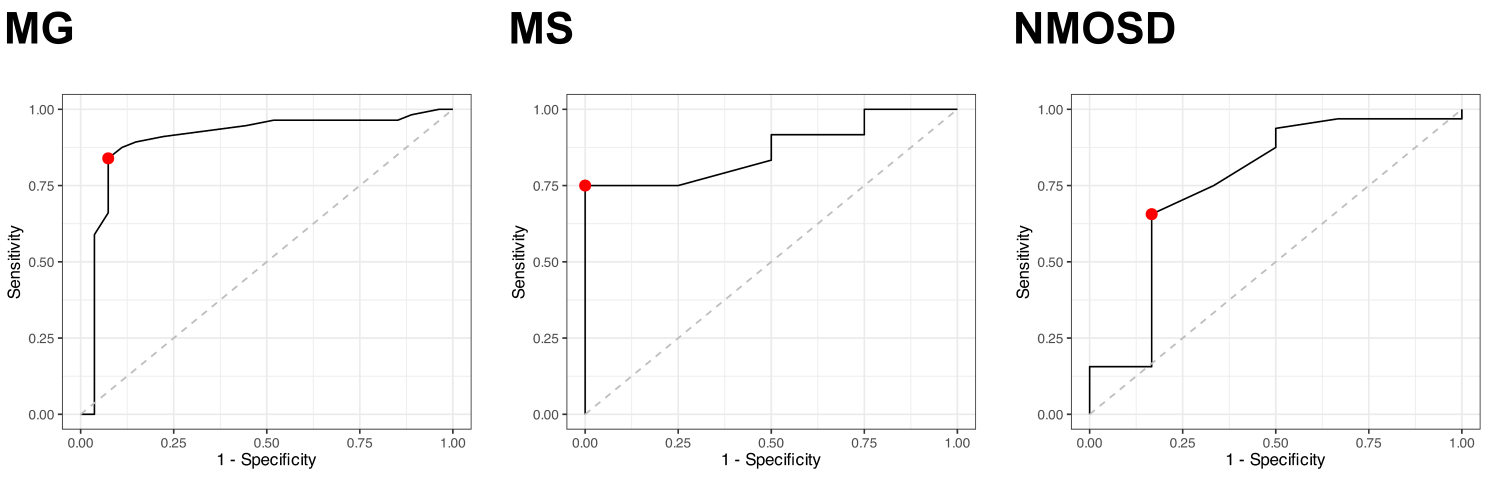


**Supplementary Figure 7** ROC Curves for MG, MS and NMOSD Subgroups

# Supplementary Tables

**Supplementary Table 1** Questionnaire on Medication Adherence and Illness Perception among Patients with NIDs

**Supplementary Table 2** Validation Results for ROC Curves

**Supplementary Table 3** Criterion Validity for MMAS-8

**Supplementary Table 4** Criterion Validity for BIPQ

## **Supplementary Table 1** Questionnaire on Medication Adherence and Illness Perception among Patients with NIDs

## **1 General Information**

| **Name/Patient No.** |  | **Sex** | ○Female ○Male | **Age** |  |
| --- | --- | --- | --- | --- | --- |
| 1. **Your educational background** | | | | | |
| ○High school or below ○Above high school | | | | | |
| **2. Your household size** | | | | | |
| ○Living alone ○Living with 2-5 people ○Living with more than 5 people | | | | | |
| **3. Your residential area** | | | | | |
| ○Urban ○Town ○Rural | | | | | |
| **4. Your average monthly income (including minors)** | | | | | |
| ○≤￥3000 ○＞￥3000 | | | | | |
| **5. Your insurance (multiple choices allowed)** | | | | | |
| ○No insurance purchased ○Mandatory natioanl insurance ○Commercial insurance | | | | | |

**2 Eight-Item Morisky Medication Adherence Scale, MMAS-8**

| **Items** | **Options** | |
| --- | --- | --- |
| 1. Do you sometimes forget to take your medicine? | ○Yes  (0 point) | ○No  (1 point) |
| 2. In the past two weeks, have you forgotten to take your medicine for one or more days? | ○Yes  (0 point) | ○No  (1 point) |
| 3. During treatment, when you feel your symptoms worsen or other symptoms appear, do you reduce the dosage without informing your doctor? | ○Yes  (0 point) | ○No  (1 point) |
| 4. When you travel or are away from home for a long time, do you sometimes forget to take your medicine with you? | ○Yes  (0 point) | ○No  (1 point) |
| 5. Did you take your medicine yesterday? | ○Yes  (1 point) | ○No  (0 point) |
| 6. When you feel your symptoms have improved or disappeared, have you stopped taking your medicine? | ○Yes  (0 point) | ○No  (1 point) |
| 7. Do you find it difficult to adhere to the treatment plan? | ○Yes  (0 point) | ○No  (1 point) |
| 8. Do you find it hard to remember to take your medicine on time and in the correct dosage? | ○Never (1 point)  ○Occasionally (0.75 points)  ○Sometimes (0.5 points)  ○Often (0.25 points)  ○All the time (0 point) | |
| **Total Score** |  | |

**3 The Brief Illness Perception Questionnaire, BIPQ**

| *Please circle a number on the scale to indicate how strongly you agree or disagree with each of the following statements.* |
| --- |
| **1. How much does your illness affect your life?** |
| 0 1 2 3 4 5 6 7 8 9 10 |
| *No impact at all*  *Severely affects my life* |
| **2. How long do you think your illness will last?** |
| 0 1 2 3 4 5 6 7 8 9 10 |
| *A very short time*   *Forever* |
| **3. To what extent do you feel you can control your illness?** |
| 0 1 2 3 4 5 6 7 8 9 10 |
| *Absolutely no control Extremely high control* |
| **4. To what extent do you think the treatment you are receiving (medication, etc.) will help your illness?** |
| 0 1 2 3 4 5 6 7 8 9 10 |
| *No help at all Extremely helpful* |
| **5. How severe are the symptoms caused by your illness?** |
| 0 1 2 3 4 5 6 7 8 9 10 |
| *No symptoms at all Many severe symptoms* |
| **6. How concerned are you about your illness?** |
| 0 1 2 3 4 5 6 7 8 9 10 |
| *Not concerned at all Extremely concerned* |
| **7. To what extent do you feel you understand your illness?** |
| 0 1 2 3 4 5 6 7 8 9 10 |
| *No understanding at all Clearly understand* |
| **8. How much does the illness affect your emotions? (e.g., does it make you angry, scared, depressed, or melancholy?)** |
| 0 1 2 3 4 5 6 7 8 9 10 |
| *No emotional impact at all Extremely emotionally affected* |
| **9. Please list the three most important factors you believe caused your illness in order of importance; among them, the most important cause of illness in your opinion is：** |
| 1. 2. 3. |

**Supplementary Table 2** Validation Results for ROC Curves

|  | MG | MS | NMOSD |
| --- | --- | --- | --- |
| Sensitivity | 0.839 | 0.750 | 0.656 |
| Specificity | 0.926 | 1.000 | 0.833 |
| PPV | 0.959 | 1.000 | 0.955 |
| NPV | 0.735 | 0.571 | 0.312 |
| AUC | 0.899 | 0.865 | 0.760 |
| 95% of AUC | 0.813-0.986 | 0.680-1.000 | 0.499-1.000 |
| Optimal Cutoff Value | 51.5 | 52 | 52.5 |

**Supplementary Table 3** Criterion Validity for MMAS-8

| **MG** | Unstandardized Coefficient | | | β | *t* | *P* |
| --- | --- | --- | --- | --- | --- | --- |
|  | B | SE | 95%CI |  |  |  |
| Gender | -0.228 | 0.373 | [-0.970,0.514] | -0.073 | -0.611 | -0.543 |
| Age | -0.008 | 0.011 | [-0.031,0.014] | -0.086 | -0.727 | -0.469 |
| Education | -0.250 | 0.372 | [-0.990,0.491] | -0.081 | -0.671 | -0.504 |

| **MS** | Unstandardized Coefficient | | | β | *t* | *P* |
| --- | --- | --- | --- | --- | --- | --- |
|  | B | SE | 95%CI |  |  |  |
| Gender | -0.052 | 0.753 | [-1.694,1.590] | -0.017 | -0.069 | 0.946 |
| Age | -0.026 | 0.039 | [-0.112,0.059] | -0.168 | -0.672 | 0.515 |
| Education | 1.857 | 0.704 | [0.323,3.390] | 0.636 | 2.638 | 0.022 |

| **NMOSD** | Unstandardized Coefficient | | | β | *t* | *P* |
| --- | --- | --- | --- | --- | --- | --- |
|  | B | SE | 95%CI |  |  |  |
| Gender | -0.171 | 0.801 | [-1.799,1.457] | -0.042 | -0.214 | 0.832 |
| Age | 0.011 | 0.017 | [-0.024,0.046] | 0.122 | 0.627 | 0.535 |
| Education | 0.420 | 0.631 | [-0.862,1.702] | 0.135 | 0.665 | 0.510 |

**Supplementary Table 4** Criterion Validity for BIPQ

| **MG** | Unstandardized Coefficient | | | β | *t* | *P* |
| --- | --- | --- | --- | --- | --- | --- |
|  | B | SE | 95%CI |  |  |  |
| Gender | 7.744 | 5.743 | [-3.928,19.415] | 0.259 | 1.348 | 0.186 |
| Age | 0.083 | 0.125 | [-0.170,0.337] | 0.125 | 0.666 | 0.510 |
| Education | 3.607 | 4.523 | [-5.584,12.798] | 0.157 | 0.797 | 0.431 |

| **MS** | Unstandardized Coefficient | | | β | *t* | *P* |
| --- | --- | --- | --- | --- | --- | --- |
|  | B | SE | 95%CI |  |  |  |
| Gender | 7.683 | 4.944 | [-3.089,18.454] | 0.368 | 1.554 | 0.146 |
| Age | 0.260 | 0.258 | [-0.302,0.822] | 0.248 | 1.007 | 0.334 |
| Education | -8.397 | -0.431 | [-18.460,1.666] | -0.431 | -1.818 | 0.094 |

| **NMOSD** | Unstandardized Coefficient | | | β | *t* | *P* |
| --- | --- | --- | --- | --- | --- | --- |
|  | B | SE | 95%CI |  |  |  |
| Gender | -0.171 | 0.801 | [-1.799,1.457] | -0.042 | -0.214 | 0.832 |
| Age | 0.011 | 0.017 | [-0.024,0.046] | 0.122 | 0.627 | 0.535 |
| Education | 0.420 | 0.631 | [-0.862,1.702] | 0.135 | 0.665 | 0.510 |
